# Automated home monitoring and management of patient‐reported symptoms during chemotherapy: results of the symptom care at home RCT

**DOI:** 10.1002/cam4.1002

**Published:** 2017-01-30

**Authors:** Kathi H. Mooney, Susan L. Beck, Bob Wong, William Dunson, Debra Wujcik, Meagan Whisenant, Gary Donaldson

**Affiliations:** ^1^ Huntsman Cancer InstituteUniversity of UtahCollege of NursingSalt Lake CityUtah

**Keywords:** Chemotherapy, decision support systems, eHealth, evidence‐based clinical practice guidelines, patient‐reported outcomes, symptom management, telehealth

## Abstract

Technology‐aided remote interventions for poorly controlled symptoms may improve cancer symptom outcomes. In a randomized controlled trial, the efficacy of an automated symptom management system was tested to determine if it reduced chemotherapy‐related symptoms. Prospectively, 358 patients beginning chemotherapy were randomized to the Symptom Care at Home (SCH) intervention (*n* = 180) or enhanced usual care (UC) (*n* = 178). Participants called the automated monitoring system daily reporting severity of 11 symptoms. SCH participants received automated self‐management coaching and nurse practitioner (NP) telephone follow‐up for poorly controlled symptoms. NPs used a guideline‐based decision support system. Primary endpoints were symptom severity across all symptoms, and the number of severe, moderate, mild, and no symptom days. A secondary endpoint was individual symptom severity. Mixed effects linear modeling and negative binominal regressions were used to compare SCH with UC. SCH participants had significantly less symptom severity across all symptoms (*P* < 0.001). On average, the relative symptom burden reduction for SCH participants was 3.59 severity points (*P* < 0.001), roughly 43% of UC. With a very rapid treatment benefit, SCH participants had significant reductions in severe (67% less) and moderate (39% less) symptom days compared with UC (both *P* < 0.001). All individual symptoms, except diarrhea, were significantly lower for SCH participants (*P* < 0.05). Symptom Care at Home dramatically improved symptom outcomes. These results demonstrate that symptoms can be improved through automated home monitoring and follow‐up to intensify care for poorly controlled symptoms.

## Introduction

Many studies have shown the feasibility of electronic capture and monitoring of patient‐reported outcomes (PROs) for symptoms related to cancer treatment 1, 2, 3, 4, 5, 6, 7, 8, 9, 10, 11, 12, 13, 14. Most studies have focused on PRO capture at clinic visits with fewer studies evaluating automated systems developed for between‐visit home monitoring and real‐time follow‐up for intensification of care for poorly controlled symptoms. Studies have documented feasibility and high patient adherence and satisfaction, but recent reviews conclude that there is little evidence of impact on health outcomes 15, 16, 17, 18. Recently, Basch et al. (2016) found that participants randomized to daily automated symptom reporting with subsequent provider alerts, experienced better health‐related quality of life compared to usual care participants 19. Few other studies have reported symptom outcomes and those that have, demonstrate mixed results 5, 7, 11, 13, 14, 19, 20, 21. Also rarely reported is how oncology providers act upon electronically provided PRO information, although there is evidence that clinic visit discussions of symptoms are increased 2, 11, 16, 18.

Automated systems facilitate home symptom monitoring between scheduled clinic visits. Acute chemotherapy symptoms typically manifest during the interim period, and waiting to capture PROs at clinic visits misses peak symptom distress. Poorly controlled symptoms can lead to unplanned clinic or emergency department visits and unplanned hospitalizations 22.

We developed and tested an automated system for remotely monitoring chemotherapy symptoms between visits 10, 11. The initial telephone‐based interactive voice response system included daily home monitoring of chemotherapy symptoms with provider alerts for poorly controlled symptoms. Our first randomized trial, like other studies, demonstrated high patient satisfaction and usability, but not improvement in symptom outcomes 11. Interestingly, we found that oncology providers rarely followed up on alerts. Of the 1028 alerts, 457 reported a symptom severity for one or more symptoms of eight or greater on a 10‐point scale, yet there were only 20 provider‐initiated contacts. In subsequent interviews, providers voiced barriers to follow‐up that were consistent with two concepts that underlie clinical inertia: clinical uncertainty about the value of further treatment and competing demands 23, 24, 25. They believed the best symptom treatment was provided at chemotherapy initiation and there was little improvement to be gained in altering treatment. They also cited health system barriers, including lack of time and inadequate reimbursement for follow‐up 11.

We report here the findings of our subsequent randomized controlled trial to specifically address provider beliefs that symptom care intensification is unlikely to improve outcomes. We developed Symptom Care at Home (SCH) utilizing the original interactive voice response system, but expanded it to include four components: automated daily monitoring of 11 chemotherapy‐related symptoms, automated self‐management coaching tailored to the reported symptom pattern, automated alerts for poorly controlled symptoms, and an electronic symptom guideline‐based decision support system for use by study‐based nurse practitioners (NP) who consistently provided telephone follow‐up to intensify care for alerting symptoms. The study objectives were to determine if the SCH intervention significantly decreased symptom severity overall and for the 11 individual symptoms monitored and whether it decreased days where the highest symptom(s) was reported at moderate or severe intensity and increased days of no or mild symptom intensity as compared to enhanced usual care (UC).

## Methods

### Study design and participants

A longitudinal randomized clinical trial was conducted with equal allocation assignment to the SCH intervention or UC. The automated system collected patient‐reported data from all participants on the presence and severity of 11 symptoms: fatigue, trouble sleeping, nausea and vomiting, pain, numbness or tingling, feeling blue or down, feeling nervous or anxious, distress over appearance, diarrhea, sore mouth, and trouble thinking or concentrating.

#### Eligibility and enrollment of participants

Patients were recruited from four oncology practices at a cancer center in the Intermountain West and two oncology practices at a public hospital associated with a comprehensive cancer center partnership in the South, both of which had clinical trials offices and routine participation in research efforts. Inclusion criteria included those: 18 years or older, life expectancy of at least 3 months, beginning a cancer chemotherapy course planned for at least three cycles, English speaking, with daily access to a telephone. Exclusions included concurrent radiation therapy and treatment that was exclusively biotherapy. IRB approval was obtained from the required boards and the trial was registered on ClinicalTrials.gov (NCT01973946). Patients were randomly assigned by the statistician to the SCH intervention or to UC in blocks of 10 for each of the six provider practices using the sequentially numbered opaque envelope method.

Potential study participants were identified and approached at their treatment planning visit. Research staff obtained informed consent, then demographic information, then opened the envelope to determine group assignment, and trained the participant in the telephone reporting system. Participants were trained identically, with the exception that participants assigned to the SCH group were instructed that symptoms reported at or above the threshold level would alert a study NP. Both UC and SCH participants were told that they would be reminded on each call to notify their oncology provider with concerns about symptoms. UC participants were told that symptom data they reported to the automated phone system would not be seen by anyone.

All participants reported symptom data during a daily phone call with the automated system. During this call, participants were queried about the presence of each symptom and if the symptom was present, they were asked to rate the severity on a scale of 1–10 (10 being most severe). Questions were asked verbally by the automated system and answered by participants using the touch‐tone keypad. The automated interactive voice response system is further described in our previous report 11. Single‐item measures are commonly used and accepted for clinically reporting the severity associated with patient‐reported symptoms 11, 26.

### Intervention and usual care conditions

#### SCH intervention group

The SCH system monitored daily, patient‐reported symptom data, and immediately provided automated algorithm‐based self‐care management messages tailored to the reported symptom prevalence and severity. The messages were based on national evidence‐based guidelines and validated by a national panel of symptom experts. Algorithms vary coaching content based on severity level (mild, moderate, or severe), contextual responses (e.g., # of times vomited, low fluid intake), whether it was a new symptom or reported previously, and was getting better or worse. Messages also had decision rules about when and how often to play.

At the end of the call, the SCH system generated automated alerts for symptoms that exceeded preset thresholds to the web‐based SCH decision support system (DSS) for NP follow‐up. For the 11 symptoms, 29 different responses generated an alert; for example, a severity rating of 5 or greater (1–10 scale) or a pattern of responses such as fatigue at a level of 4 or greater in 3 of the past 7 days.

Study‐based NPs managed alerts with DSS guidance by telephoning intervention participants to provide intensified symptom care. Participants were asked to call by noon each day and 7 days a week, NPs returned calls to SCH participants with alerts in a 4‐h period each afternoon. If the participant was unavailable to receive the NP call, a message was left and the NP would try later during the 4‐h call window. To protocolize the care, SCH integrated three systems: the SCH call‐generated symptom alerts, the SCH DSS, and access to the patient's electronic health record (EHR) providing key organizing functions including graphing the patient's historical symptom severity ratings, providing access to laboratory or radiology reports, displaying alerting symptom guideline‐based follow‐up assessments, pharmacological and nonpharmacological interventions, and possible referrals and providing links to the actual national guidelines for the symptom.

The primary source for the DSS symptom guidelines were the National Comprehensive Cancer Network supportive care guidelines. Other sources included the American Society of Clinical Oncology, Multinational Association of Supportive Care in Cancer, the Oncology Nursing Society and Cancer Care Ontario. The NPs had prescriptive privilege and wrote prescriptions based on the national guidelines after review by participating physicians and the clinical pharmacist at one of the participating sites. In addition, NPs recorded all their actions in the patient's electronic health record and also emailed the responsible oncologist about interactions with their patients including altered medication doses or new prescriptions.

We systematically audited NP utilization of the DSS and audio taped a random sample of calls to audit guideline adherence, holding monthly meetings to discuss audit findings and maintain intervention fidelity. A more detailed report of the SCH system is reported elsewhere (Beck et al. under review) 27.

#### Enhanced usual care control group

The enhanced usual care attentional control group called the automated symptom reporting system daily and reported presence and severity of the 11 symptoms. They did not receive self‐management coaching and poorly controlled symptoms did not alert the study NPs. At study entry and on every automated call, participants were reminded to call their provider for symptom concerns. We considered the control group to be enhanced usual care because the UC participants reported their symptoms on a daily basis and were reminded to call their provider for concerns.

### Timing of assessments and study measures

Patient‐reported demographics and disease‐related characteristics were collected at baseline. Patient‐reported presence and severity of 11 symptoms were collected daily from study entry through chemotherapy course completion or 6 months, whichever came first.

### Statistical analysis

An initial power analysis via longitudinal mixed modeling was conducted utilizing the previous study's data. A total of 280 participants (140 in each group) showed 80% power to detect a standardized effect size of 0.50 with an alpha = 0.05. The primary endpoints were symptom severity across symptoms and the number of severe, moderate, mild, and no symptom days. A secondary outcome was individual symptom severity. The trial ended when the sample size was achieved. There were no reported harms or unintended effects.

#### Descriptive data

Descriptive data (sociodemographic and disease characteristics) were compared between groups examining cross‐classifications for categorical variables and means, standard deviations, and skew for continuous variables. Pearson Chi‐square for categorical variables and independent *t*‐tests for continuous variables were conducted for covariates, though principal analyses controlled for any randomization imbalance by conditioning on baseline values.

#### Overall symptom severity

Overall symptom severity, defined as the sum of 0–10 severity ratings across symptoms reports, was analyzed using mixed effects linear models as a function of time on study and group assignment. In this full intent‐to‐treat analysis, we incorporated all postrandomization observations on each person, conditioning on prerandomization covariates, allowing for mean and individual differences in level (i.e., fixed and random postrandomization intercepts), and mean rate‐of‐change (slope). In this model, with baseline conditioning, both parameters convey information about treatment impact. In the event of treatment arm differences in rate‐of‐change, the expected impact changes over time. We therefore also calculated the expected difference at key time points (adjusted for baseline). These differences represent the expected treatment difference at various follow‐up points for patients statistically matched on prerandomization baseline scores. Following the definitive test of treatment impact as a function of random assignment only, we examined whether conditioning on the key covariates of gender, age, diagnosis, and their interactions with group and time qualified any aspect of this impact. To minimize the risk of capitalizing on chance relationships, we accepted a more complex model only if the added terms improved the Bayesian information criterion.

#### Number of days with severe, moderate, mild, and no symptoms

The highly skewed nature of time on study required transformation of the simple linear metric for time. The overall symptom analysis, described in the previous section, maintained approximate linearity by taking the square root of time as the dependent variable. In an independent approach, we aggregated the response into ordered categories commonly used in evaluating symptom severity (no [0], mild [1–3], moderate [4–7], and severe [8–10]), and modeled the relative frequency of occurrence over time 28. Due to overdispersion of the data, negative binomial regression was used to investigate how the number of severe, moderate, mild, and no symptom days across all symptoms varied by treatment. Separate models were built for number of days of highest reported symptom(s) intensity. Alpha level was set at 0.05 for all tests.

## Results

**Figure 1:**
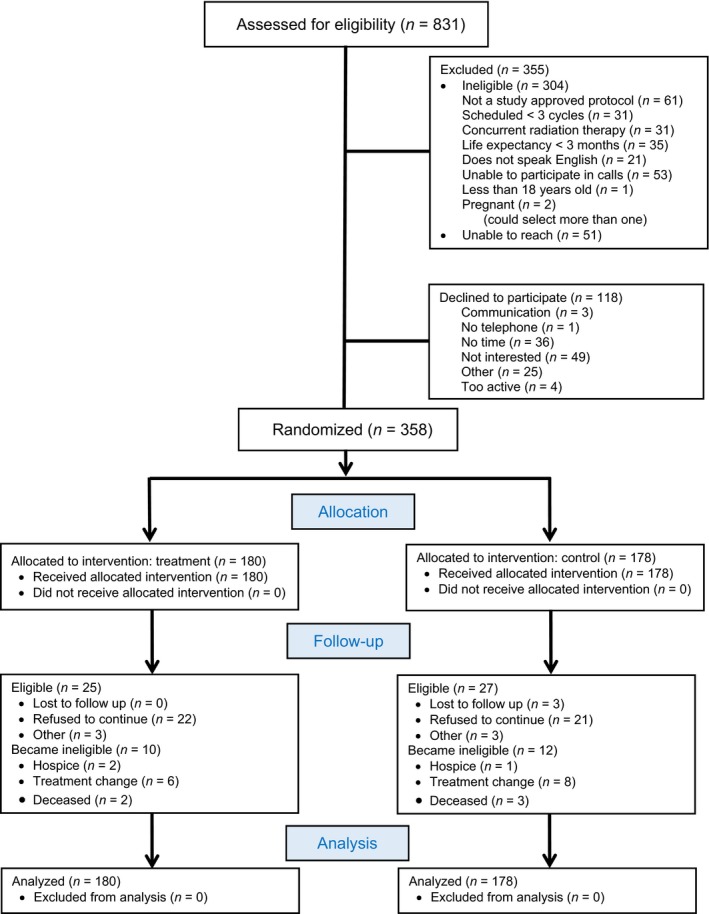
CONSORT 1 Flow Diagram

Among the 831 patients screened, 304 did not meet inclusion criteria and 51 were unable to reach (Fig. 1). We approached 476 eligible individuals; 358 (75.2%) agreed to participate and were randomized to the SCH group (180) or the UC group (178). Participant average age was 55.8 years (SD = 11.42), with the majority female (*n* = 270, 75.4%), White/Caucasian (*n* = 297, 83.0%), with breast cancer (*n* = 156, 43.6%) or lung cancer (*n* = 61, 17.0%). There were no significant differences between groups for demographic or disease variables and symptom severity at baseline (Table 1).

**Table 1:** Participant's characteristics

Characteristics	UC^a^ (*n* = 178)	SCH^a^ (*n* = 180)	All (*n* = 358)	*P*‐value
	M (SD)	M (SD)	M (SD)	
Age				
	56.79 (10.54)	54.77 (12.17)	55.77 (11.42)	0.10
	*n* (%)	*n* (%)	*n* (%)	
Gender				0.85
Female	135 (76)	135 (75)	270 (75)	
Male	43 (24)	45 (25)	88 (25)	
Ethnicity				0.47
Non‐Hispanic	171 (96)	170 (94)	341 (95)	
Hispanic	7 (4)	10 (6)	17 (5)	
Race				0.25
White	154 (86)	143 (80)	297 (83)	
Black	19 (11)	22 (12)	41 (12)	
Asian	2 (1)	3 (2)	5 (1)	
Native American	1 (1)	4 (2)	5 (1)	
Other/unknown	2 (1)	8 (4)	10 (3)	
Marital status				0.91
Married or living with a partner	116 (65)	117 (65)	233 (65)	
Single	32 (18)	30 (17)	62 (17)	
Other (divorced, separated, widowed)	30 (17)	33 (18)	63 (18)	
Education				0.24
Less than high school graduate	18 (10)	10 (5)	28 (8)	
High school graduate/GED	37 (21)	46 (26)	83 (24)	
Some college/technical school or Associate's degree	60 (34)	70 (39)	130 (36)	
Bachelor's degree	38 (21)	28 (16)	66 (18)	
Postgraduate education	25 (14)	26 (14)	51 (14)	
Annual Household Income				0.45
Less than $19,999	34 (19)	38 (21)	72 (20)	
$20,000–49,999	47 (26)	44 (25)	91 (25)	
$50,000–69,999	19 (11)	20 (11)	39 (11)	
$70,000 and higher	61 (34)	64 (36)	125 (35)	
Declined to answer	17 (10)	13 (7)	30 (9)	
Employment				0.09
Not employed outside the home	60 (34)	77 (43)	137 (38)	
Employed part or full‐time	54 (30)	56 (31)	110 (31)	
Other (Retired, sick leave, or disability)	64 (36)	47 (26)	111 (31)	
Cancer diagnosis				0.50
Breast	73 (41)	83 (46)	156 (44)	
Lung	28 (16)	33 (18)	61 (17)	
Ovarian	15 (9)	21 (12)	36 (10)	
Colorectal	16 (9)	10 (6)	26 (7)	
Pancreatic	11 (6)	10 (6)	21 (6)	
Head and neck	7 (4)	6 (3)	13 (4)	
Endometrial	6 (3)	3 (2)	9 (3)	
Other	22 (12)	14 (8)	36 (10)	
Cancer stage				0.11
I	20 (11)	21 (12)	41 (11)	
II	28 (16)	45 (25)	73 (20)	
III	47 (26)	34 (19)	81 (23)	
IV	83 (47)	80 (44)	163 (46)	

a Symptom Care at Home Intervention Group (SCH), Usual Care (UC).

Daily call adherence was high with no difference between groups (*P* = 0.80); on average, participants made 90% of expected calls and were on study for 77 days. Average length of calls was 4:45 min for SCH and 4:19 min for UC. Study NPs completed 1756 alert follow‐up calls to SCH intervention participants with an average call length of 7 min. UC participants were instructed on each call to contact their providers if they had symptoms concerns, they did this only 5% of the time when reporting one or more symptoms at 4 or greater on the 10‐point scale.

There was no difference in noncompletion rates between groups, with 12% voluntary withdrawal, and 2.5% who were withdrawn because they were either no longer receiving chemotherapy, added radiation to their treatment plan, or changed care to a physician not located at one of our participating clinics. The noncompleters (*n* = 25 SCH, *n* = 27 UC) were slightly older (58.45 vs. 55.08 years, *P* = 0.02**)** and more likely to have lung cancer (35% vs. 12%, *P* < 0.01).

Symptoms were common, with fatigue as the most prevalent, reported at moderate‐to‐severe levels by 86% of participants, followed by pain (80%), trouble sleeping (78%), and nausea (60%) (Table 2). UC participants reported symptoms that fit alerting patterns for 37% of calls, whereas SCH participants alerted on 19% of calls.

**Table 2:** Prevalence of symptoms reported at moderate or severe levels one or more days

Symptoms	% (*n* = 358)
Fatigue	86
Pain	80
Trouble sleeping	78
Nausea/vomiting	60
Depressed mood	52
Feeling nervous/anxious	49
Trouble thinking/concentrating	48
Numbness/tingling	42
Diarrhea	38
Sore mouth	38
Concern with changes in appearance	34

**Figure 2:**
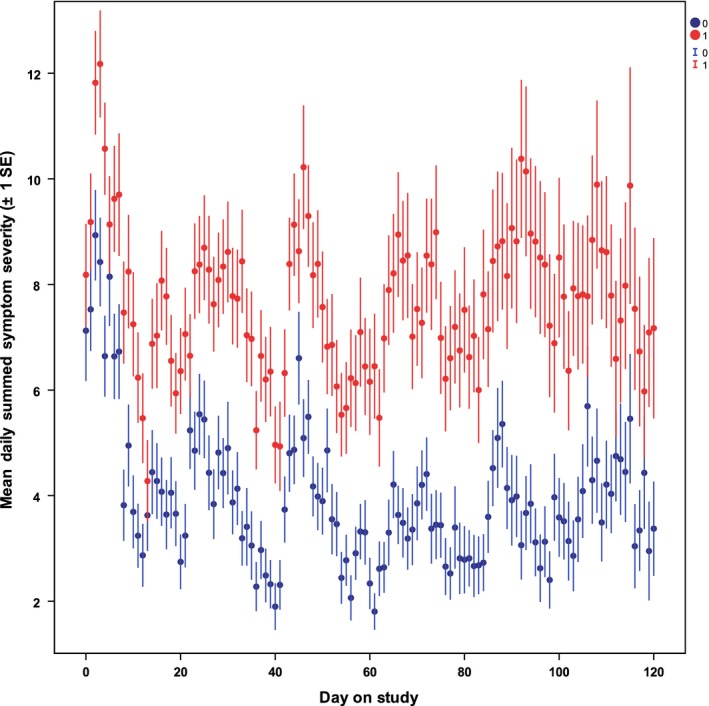
Daily values for total symptom severity summed across all 11 symptoms

Figure 2 plots mean daily values for overall symptom severity (summed across all symptoms) for the first 4 months on study. The mean last observations were 87 days and 89 days, for UC and SCH‐Hospice intervention, respectively; 75th percentiles were 124 days for both and box plots of last observations were indistinguishable. While Figure 2 shows data out to 4 months only because confidence intervals widen as sample sizes diminish, the analyses used data from all time values. The mean data show a rapid treatment benefit, with SCH participants, after 1 week on study, consistently reporting approximately half the overall severity of UC participants. Inferential results are based on the intent‐to‐treat mixed effects analysis, which retains all observations while adjusting for baseline imbalance (Table 3).

**Table 3:** Intent‐to‐treat maximum‐likelihood mixed effects analysis^a^

Effect	Coefficient	SE	*t* or Wald	*P* for likelihood‐ratio test
Fixed				
Regression on baseline^b^	0.276	0.035	7.96	<0.001
Treatment impact^c^	3.589	0.721	4.98	<0.001
Adjusted means^d^				
UC (*n* = 178)	8.384	0.510	17.32	<0.001
SCH (*n* = 180)	4.795	0.509	9.420	<0.001
Adjusted treatment impact				
Day 7	2.95	0.734	4.02	<0.001
Day 30	3.41	0.721	4.72	<0.001
Day 60	3.77	0.724	5.21	<0.001
Day 90	4.05	0.736	5.51	<0.001
Day 120	4.28	0.750	5.71	<0.001
Treatment Impact on rate‐of‐change^e^	0.1607	0.042	3.827	<0.001
Rate within arm^f^				
UC^i^ (*n* = 178)	−0.0077	0.031	−0.254	0.799
SCH^i^ (*n* = 180)	−0.1684	0.029	−5.747	<0.001
Random				
Individual gain^g^	Var = 29.86 SD = 5.46	0.46	64.91	<0.001 (Wald)
Within‐person residual^h^	Var = 43.44 SD = 6.59	3.00	14.48	<0.001 (Wald)
Intraclass correlation	0.41			

a All observations, analyzing square root of study day to reduce impact of positive skew.

b Regression of outcome on baseline, assumed equal across groups.

c Primary endpoint contrast: Overall treatment impact estimated as the difference in baseline‐adjusted symptom means evaluated at the overall means of the covariates (the sample mean square root of study day).

d Estimated postrandomization adjusted symptom means (evaluated at mean square root of study day).

e Difference in mean rate‐of‐change between groups.

f Within‐treatment arm rate‐of‐change expressed as expected change in symptom severity per unit increase in square root of study day.

g Model‐inferred variation in systematic individual symptom change from baseline.

h Nonsystematic variation about systematic fixed and individual effects.

i SCH, Symptom Care at Home Intervention Group; UC, Usual Care.

On average, the treatment impact (postbaseline symptom burden reduction) for SCH participants was 3.59 severity points (*P* < 0.001), roughly 43% of the UC value. The average rate‐of‐change for SCH participants significantly (*P* < 0.001) improved by 0.161 symptoms points per square root of day on study when compared to UC, with a SHC mean slope of −0.169 (*P* < 0.001) versus the UC mean slope of −0.008 (*P* = 0.799). Since the arm‐by‐time interaction was significant, the specific treatment impact varied by duration on study, ranging from 2.95 to 4.28 symptom points at key assessment times, with all comparisons significant (*P* < 0.001). The mixed effects longitudinal model distinguishes typical systematic differences in gain or loss across people (SD = 5.46) from measurement and other error within people (SD = 6.59), a partition allowing more precise characterization of relative effect magnitudes. Compared to systematic individual differences, effect sizes at key time points range from 0.55 to 1.02. No covariate or combination of explanatory covariates improved the Bayesian information criterion.

In a complementary analytical approach, we applied negative binomial regression in a direct nonlinear model of skewed, infrequent, overdispersed data. We then investigated the SCH intervention's effect on highest symptom(s) severity/day (severe: ≥8; moderate: 4–7; mild: 1–3; none: 0). SCH participants had three times fewer (67% less) severe days (*P* < 0.001) and 1.65 times fewer (39% less) moderate days (*P* = 0.001) than UC. SCH had 39% more mild days (*P* = 0.016) and 25% more no symptom days (*P* = 0.006) than UC (Table 4).

**Table 4:** Mean estimates and odds ratios from negative binomial regression modeling of highest symptom severity days

	UC^a^M (SD) (*n* = 178)	SCH^a^ M (SD) (*n* = 180)	OR [95% CI]	*P*‐value
Total reporting days	76.24 (3.33)	77.43 (3.23)	0.99 [0.88–1.11]	0.797
Severe days (8–10)	16.93 (2.33)	5.64 (0.67)	3.00 [2.10–4.29]	< 0.001
Moderate days (4–7)	21.81 (2.20)	13.20 (1.39)	1.65 [1.24–2.20]	0.001
Mild days (1–3)	10.59 (1.46)	17.42 (2.69)	0.61 [0.41–0.91]	0.016
No symptom days (0)	47.54 (3.93)	63.05 (3.83)	0.75 [0.62–0.92]	0.006

a Symptom Care at Home Intervention Group (SCH), Usual Care (UC).

Ten of the 11 symptoms were significantly lower for SCH participants (*P*: 0.025 to < 0.001) than UC (Table 5). Diarrhea, less commonly reported, was not significantly different from UC.

**Table 5:** Estimated mean difference of symptom severity between SCH^b^ (*n* = 180) and UC^b^ (*n* = 178) groups^a^

	Estimated mean difference (SCH^b^ – UC^b^)^a^	SEM	df	t	*P*‐value
Fatigue severity	−0.685	0.167	324.688	−4.107	<0.001
Pain severity	−0.605	0.164	320.533	−3.691	<0.001
Trouble sleeping severity	−0.281	0.0962	277.144	−2.920	0.004
Nausea/vomiting severity	−0.291	0.105	295.69	−2.769	0.006
Depressed mood severity	−0.241	0.085	247.62	−2.850	0.005
Feeling nervous/anxious severity	−0.206	0.092	273.081	−2.247	0.025
Trouble thinking/concentrating severity	−0.319	0.115	287.657	−2.775	0.006
Numbness/tingling severity	−0.329	0.107	346.996	−3.067	0.002
Diarrhea severity	−0.081	0.047	248.547	−1.732	0.085
Sore mouth severity	−0.257	0.081	282.720	−3.172	0.002
Concern with changes in appearance severity	−0.219	0.072	297.663	−3.039	0.003

a Negative value indicates decreased symptom severity compared to UC.

b Symptom Care at Home Intervention Group (SCH), Usual Care (UC).

## Discussion

When daily automated symptom monitoring, self‐management coaching, and NP follow‐up using guideline‐based decision support were combined for between‐visit care, we found significant reductions in symptom burden overall, for 10 of 11 symptoms individually, and for categorical symptom days. These reductions were both statistically and clinically significant and contrast with our previous study where oncology providers did not intensify symptom care 11. This suggests that one major barrier to better symptom management may be provider inaction and clinical inertia. Clinical inertia is a provider response reported in hypertension, hyperlipidemia, and diabetic control literature but had not previously been reported for cancer‐related symptom treatment 23, 24, 25. In our previous study, providers stated that they were uncertain if symptom intensification improved symptom outcomes. This study provides strong evidence that symptom care intensification by telephone, utilizing supportive care guidelines and self‐management coaching, can yield significant reduction in symptom burden and is efficacious.

Besides provider response to unrelieved symptoms, availability and use of symptom guidelines may be important to achieving symptom reduction. A recent study examined patients with advanced lung cancer, where weekly automated symptom monitoring and provider alerts failed to reduce symptom burden 14. Providers were alerted and nurses contacted patients within 1 day but did not use guideline‐based decision support. A combination of infrequent weekly monitoring and no symptom guidelines may explain the difference in our results.

Our study involved multiple cancers and disease stages and monitored a variety of symptoms, providing evidence that remote automated symptom care is appropriate for chemotherapy care broadly. Given et al. 20, 21 reported that automated monitoring with tailored instructions was as effective as a nurse‐delivered phone intervention for multiple cancers and symptoms and was more effective than the nurse‐delivered intervention among patients with metastatic disease.

Remote monitoring and intensified care of symptoms at home extends cancer care to patients where they live and pairs care with real‐time symptom needs. Automated systems provide efficiencies and judicious use of provider time in follow‐up. It also provides an extension of care availability for patients living at a distance from treatment centers and is particularly important for those living in rural communities where supportive care services are geographically limited.

Our findings have some restrictions due to limited diversity. Our sample was predominantly female and White, however, we had 12% African American participation. The automated intervention has not been tested in languages other than English.

Another limitation is our inability to determine the active ingredient(s) of our multicomponent intervention, thus, it is unknown what the minimum necessary components are to achieve significant reduction in symptom severity. Others have emphasized the importance of examining the components of multicomponent interventions 21, 29.

Given the improvements we found in symptom control, we conclude that the efficacy of automated symptom monitoring is dependent on timely oncology provider response to problematic symptoms. Despite the ease of use and feasibility of remote automated monitoring, our research suggests that without timely and proper clinical follow‐up, telehealth approaches may not improve patient outcomes.

This study provides a definitive response to oncology providers who are uncertain about the value of symptom care intensification. Symptom care intensification is efficacious and benefits cancer patients with a variety of diagnoses, stages and chemotherapy regimens. Within the current health system, there remain barriers to available provider time and reimbursement for follow‐up symptom care. Movement toward a value‐based health care system may decrease current barriers and reenvision symptom care from episodic, clinic‐based point‐of‐care to continuous monitoring and management in patients’ homes, where they spend their days and experience treatment‐related symptoms.

## Conflict of Interest

There are no conflict of interest disclosures to report.
